# Autoimmune Epilepsy Temporally Associated With Lyme Disease: A Report of Two Cases

**DOI:** 10.7759/cureus.105228

**Published:** 2026-03-14

**Authors:** Pamela M Cipriano

**Affiliations:** 1 Internal Medicine, Private Practice, Torrington, USA

**Keywords:** autoimmune epilepsy, borrelia burgdorferi infection, drug-resistant epilepsy (dre), iv immunoglobulin (ivig) therapy, lyme neuroborreliosis, lyme's disease, neuroinflammation

## Abstract

The relationship between Lyme-associated illness and immune-mediated seizure disorders remains incompletely characterized. Infection-triggered neurologic immune syndromes are well described in some settings, but evidence linking *Borrelia burgdorferi* to autoimmune epilepsy remains limited and is based primarily on isolated reports. We describe two previously healthy patients, a 25-year-old male and a 14-year-old female, who developed new-onset seizures temporally associated with Lyme-associated illness and the initiation of antimicrobial therapy.

Diagnostic evaluation included serologic testing for tick-borne disease performed through outside commercial laboratories and subsequent reference laboratory testing, cerebrospinal fluid (CSF) analysis, neuroimaging, electroencephalography, neural autoantibody testing, quantitative immunoglobulins, IgG subclass testing, and complement studies. In both cases, seizure activity persisted despite multiple antiseizure medications and worsened after initiation of antimicrobial therapy. Laboratory evaluation demonstrated hypogammaglobulinemia with IgG subclass deficiencies and complement abnormalities in both cases. Neural autoantibody testing revealed elevated anti-dopamine receptor D1 antibodies in both patients; the adult patient also had a low-titer serum N-methyl-D-aspartate receptor antibody detected by a cell-based assay. Immunomodulatory therapy with systemic corticosteroids, followed by intravenous immunoglobulin, administered while antimicrobial therapy was continued, was associated with marked seizure reduction and sustained clinical improvement.

These cases demonstrate a temporal association between Lyme-associated illness, antimicrobial treatment, infection-associated immune activation, and treatment-refractory seizures. The clinical deterioration observed after initiation of antimicrobial therapy may reflect an inflammatory response associated with microbial die-off, similar to a Jarisch-Herxheimer-type reaction described in spirochetal infections. Although the findings do not establish a causal relationship between Lyme disease and autoimmune epilepsy, they raise the possibility that infection-associated immune dysregulation may contribute to seizure pathogenesis in selected patients. These observations are hypothesis-generating and support maintaining a broad differential diagnosis, including neuroimmune mechanisms, in patients presenting with new-onset seizures in the setting of suspected infectious illness.

## Introduction

Autoimmune epilepsy is increasingly recognized as a subtype of seizure disorder in which immune-mediated mechanisms contribute to neuronal dysfunction and seizure generation, often in the absence of a clearly progressive structural lesion [1,2]. Clinical features that may raise suspicion for autoimmune epilepsy include rapid onset of seizures, resistance to conventional antiseizure medications, associated neuropsychiatric or inflammatory features, detection of neuronal autoantibodies, and clinical improvement following immunotherapy [3,4]. Although autoimmune epilepsy is classically associated with well-characterized syndromes, such as anti-N-methyl-D-aspartate (anti-NMDA) receptor and LGI1 encephalitis, the spectrum of immune-mediated seizure disorders is broader and may include post-infectious or infection-associated immune activation.

Infection-triggered autoimmune neurologic syndromes are well established in some settings. For example, the association between herpes simplex virus infection and autoimmune encephalitis is well described [5]. By contrast, evidence linking *Borrelia burgdorferi* infection to autoimmune epilepsy remains limited and largely based on isolated case reports and small observational data. This distinction is important. Confirmed neuroborreliosis refers to direct neurologic involvement of *B. burgdorferi* infection, whereas the hypothesis explored in this report is different: that Lyme-associated illness, or infection-related immune activation, may contribute to an immune-mediated seizure disorder in susceptible individuals. The objective of these case reports is to describe two patients with refractory seizures occurring in temporal association with Lyme-associated illness, and to explore the potential role of infection-associated immune mechanisms in their neurologic presentations.

Lyme disease, caused by *B. burgdorferi*, is the most common vector-borne illness in the United States [6]. Standard two-tier serologic testing relies on predefined immunoblot thresholds; therefore, some specimens with reactive bands may still be reported as negative under CDC surveillance criteria [6]. For suspected neuroborreliosis, paired cerebrospinal fluid (CSF) and serum testing to determine the antibody index is recommended when appropriate, and serology is generally preferred over CSF culture or polymerase chain reaction (PCR), as direct detection of *B. burgdorferi* in CSF is uncommon and lacks sensitivity [7,8]. Importantly, CDC surveillance case definitions were developed for epidemiologic reporting and are not intended to determine whether an individual patient has Lyme disease, or to guide clinical management [9,10]. Neurologic manifestations of Lyme disease include meningitis, cranial neuropathies, radiculoneuritis, and other inflammatory neurologic syndromes [9].

Several mechanisms have been proposed through which infection may contribute to immune-mediated neurologic disease, including molecular mimicry, disruption of the blood-brain barrier, microglial activation, and post-infectious inflammatory responses [11]. Clinical worsening after initiation of antimicrobial therapy has also been described in spirochetal infections and may reflect transient inflammatory responses associated with microbial die-off, including Jarisch-Herxheimer-type phenomena [12].

In this report, we present two previously healthy patients whose seizures were refractory to multiple antiseizure medications, yet improved after immunomodulatory therapy, raising the possibility that infection-associated immune activation and neuroinflammation contributed to the observed clinical course.

## Case presentation

Case 1

A 25-year-old previously healthy male developed abrupt-onset severe panic symptoms without an identifiable psychosocial trigger. Over the following 12 months, he underwent evaluation by primary care and psychiatry. Routine laboratory testing was unrevealing, and empirical treatment with a selective serotonin reuptake inhibitor and a benzodiazepine did not improve symptoms. He resided in a Lyme-endemic region and had substantial tick exposure, prompting evaluation for tick-borne disease. Initial serologic testing was obtained by outside providers prior to referral and included enzyme immunoassay (EIA/ELISA) with reflex immunoblot analysis when indicated. These initial studies were reported as negative. During our evaluation, additional serologic testing was performed through IGeneX Laboratories. Expanded immunoblot testing demonstrated reactivity to *B. burgdorferi *antigens at 23 and 39 kDa (Table 1).

**Table 1:** Interpretation per laboratory report IGeneX criteria define positivity as reactivity to ≥2 of the following bands: 23, 31, 34, 39, 41, and 93 kDa. CDC/NYS surveillance criteria require ≥5 specified IgG bands. The reported bands in this case did not meet CDC surveillance criteria for a positive IgG immunoblot [13,14]. Results were interpreted in conjunction with the clinical presentation and exposure history.

Band (kDa)	Result
23	Positive
31	Negative
34	Negative
39	Positive
41	Negative
93	Negative

These findings met the laboratory’s interpretive criteria but did not meet CDC surveillance criteria for a positive IgG immunoblot. Accordingly, the serologic results were interpreted in conjunction with the patient’s exposure history, symptom evolution, and broader clinical evaluation rather than as a stand-alone diagnostic determinant. Approximately two weeks after initiation of antimicrobial therapy with doxycycline 100 mg twice daily, the patient experienced his first generalized tonic-clonic seizure. Seizure activity progressed to status epilepticus, requiring intubation and intensive care management. Brain MRI was unremarkable, without evidence of acute structural pathology. Serial EEG recordings did not demonstrate definitive epileptiform discharges. CSF analysis demonstrated pleocytosis (17 cells/µL) with neutrophil predominance (66%) and a markedly elevated red blood cell count (9,000 cells/µL); the specimen was cloudy and pink, with xanthochromia present (Table 2).

**Table 2:** Cerebrospinal fluid (CSF) results The elevated red blood cell count and xanthochromia are reflective of a traumatic lumbar puncture, although inflammatory change could not be excluded. Negative or nondiagnostic CSF testing does not exclude neurologic Lyme disease, as direct detection of *Borrelia burgdorferi* in CSF is uncommon and insensitive [7,8].

Parameter	Result	Reference Range
Appearance	Cloudy	Clear
Color	Pink	Colorless
Xanthochromia	Present	Absent
WBC	17 cells/µL	0-5
RBC	9,000 cells/µL	0
Neutrophils	66%	0-7%
Lymphocytes	28%	28-96%
Monocytes	4%	16-56%
Eosinophils	1%	0%
Basophils	1%	0%
Resulting Agency	Hartford Hospital	-

These CSF findings were considered potentially compatible with inflammatory change, although the elevated red blood cell count and xanthochromia also raised the possibility of a traumatic lumbar puncture. Detection of *B. burgdorferi *in CSF was not demonstrated; however, direct CSF detection is recognized to have limited sensitivity in neuroborreliosis [7,8].

Alternative causes of seizure onset were evaluated. Metabolic studies did not identify a provoking electrolyte or glucose disturbance, medication review did not reveal a clear pro-convulsant trigger, and neuroimaging did not show an acute structural lesion. Because seizures persisted despite treatment with multiple antiseizure medications - brivaracetam 100 mg twice daily, lacosamide 200 mg twice daily, cenobamate, started at 12.5 mg daily and titrated to 200 mg daily, and midazolam 5 mg nasal spray - an immune-mediated seizure disorder was considered. Neural antibody testing was ordered for further evaluation of possible autoimmune epilepsy. Serum testing revealed low-titer NMDAR antibody positivity by cell-based assay (1:10), while the reflex tissue-based immunofluorescence assay was negative (<1:240) (Table 3). No other well-validated neuronal surface or paraneoplastic antibodies were detected.

**Table 3:** Serum neural autoantibody panel Neural autoantibody testing was obtained to evaluate possible autoimmune epilepsy. Low-titer serum N-methyl-D-aspartate receptor antibody positivity, without confirmatory tissue-based immunofluorescence, should be interpreted cautiously and in the clinical context.

Antibody	Result	Method	Reference Range
NMDAR antibody	Detected (1:10 dilution)	Cell-based assay (CBA)	Negative
NMDAR IFA titer	<1:240	Tissue-based assay	<1:240
AMPA-R Ab	Negative	CBA	Negative
GABA-B-R Ab	Negative	CBA	Negative
LGI1 Ab	Negative	CBA	Negative
CASPR2 Ab	Negative	CBA	Negative
GAD65 Ab	0	Enzyme-linked immunosorbent assay (ELISA)	≤0.02 mmol/L
CRMP-5 IgG	Negative	-	Negative
ANNA-1 (Hu)	Negative	-	Negative
ANNA-2 (Ri)	Negative	-	Negative
ANNA-3	Negative	-	Negative
PCA-1 (Yo)	Negative	-	Negative
PCA-2	Negative	-	Negative
Amphiphysin Ab	Negative	-	Negative
DPPX Ab	Negative	CBA	Negative
mGluR1 Ab	Negative	Immunofluorescence assay (IFA)	Negative
Neurexin Ab	Negative	IFA	Negative

Quantitative immunoglobulin testing demonstrated hypogammaglobulinemia, with low total IgG and low IgA. IgG subclass testing showed deficiencies in subclass 1, with a level of 231, and subclass 3, with a level of 6, with subclasses 2 and 4 within range. Complement testing demonstrated a deficiency in C3, level 66, and a deficiency in C4, level 12. Collectively, these findings suggested immune dysregulation, although they were not independently diagnostic of autoimmune epilepsy. Antimicrobial therapy was escalated to intravenous ceftriaxone, 2 grams daily, and administered via a peripherally inserted central catheter (PICC) line for six weeks; oral antibiotic therapy was administered both before and after the intravenous course. Despite prolonged antimicrobial treatment and multiple antiseizure medications, seizure activity persisted.

Given the treatment-refractory clinical course and concern for an immune-mediated seizure process, the patient was treated with intravenous methylprednisolone, 1 g daily for five days, followed by intravenous immunoglobulin (IVIG). Antimicrobial therapy was continued during IVIG treatment. Seizures resolved after immunomodulatory therapy, with only transient aura-like episodes persisting for approximately two months. At six-month follow-up, he remained seizure-free on maintenance antiseizure therapy, with doxycycline 100 mg twice daily.

Case 2

A previously healthy 14-year-old female presented with new-onset myoclonic seizures shortly after returning from the Caribbean, where she reported tick and sand flea bites. Initial events consisted of brief, shock-like jerks involving both upper and lower extremities. EEG demonstrated occasional generalized irregular spikes and bursts of high-amplitude, 3-4 Hz spike-and-wave discharges lasting three to six seconds. Brain MRI showed bilateral periventricular white matter fluid-attenuated inversion recovery (FLAIR) hyperintensities, without mass effect, restricted diffusion, or susceptibility artifact. The radiology differential included inflammatory and infectious etiologies, including Lyme disease.

Initial serologic testing was obtained by outside providers and was unrevealing. Subsequent expanded immunoblot testing through IGeneX demonstrated reactivity to 23 and 39 kDa antigens (Table 4).

**Table 4:** Interpretation per laboratory report IGeneX criteria define positivity as reactivity to ≥2 of the following bands: 23, 31, 34, 39, 41, and 93 kDa. CDC/NYS surveillance criteria require ≥5 specified IgG bands. The reported bands in this case did not meet CDC surveillance criteria for a positive IgG immunoblot [13,14]. Results were interpreted within the overall clinical context.

Band (kDa)	Result
23	Positive
31	Negative
34	Negative
39	Positive
41	Negative
93	Negative

As in Case 1, these results did not meet CDC surveillance criteria for a positive IgG immunoblot, but were considered alongside the patient’s exposure history, clinical findings, and evolving neurologic course. Antiseizure therapy was initiated with levetiracetam, 750 mg twice daily. The patient was unable to tolerate the medication due to profound fatigue. Levetiracetam was decreased to 500 mg twice daily, with little improvement in her fatigue and no change in seizure frequency.

Doxycycline, 100 mg twice daily, was then started for suspected Lyme-associated illness. Within approximately two weeks of antimicrobial initiation, seizure frequency increased from roughly one event per month to several events per week. Because of worsening seizure activity despite antiseizure therapy, additional diagnostic evaluation was pursued. Neural autoantibody testing was obtained to evaluate a possible immune-mediated seizure disorder. This testing demonstrated elevated anti-dopamine receptor D1 antibodies and borderline elevations in anti-lysoganglioside GM1 and anti-tubulin antibodies, with a normal CaM kinase II value (Table 5).

**Table 5:** Neural autoantibody testing Interpretation based on laboratory reference standards.

	Anti-dopamine Receptor D1 (Titer)	Anti-dopamine Receptor D2L (titer)	Anti-lysoganglioside GM1 (titer)	Anti-tubulin (titer)	CaM Kinase II2 (% of baseline)
Patient Result	1:8000	1:2000	1:320	1:1000	100
Normal Ranges	500 to 2,000	2,000 to 8,000	80 to 320	250 to 1,000	53 to 130
Normal Mean	1.056	6,000	147	609	95
Interpretation	Elevated	Normal	Borderline	Borderline	Normal

These antibody findings were considered supportive of immune dysregulation, but were interpreted cautiously, as the clinical significance and validation of some of these markers in autoimmune epilepsy remain uncertain.

Quantitative immunoglobulin testing revealed low total IgG, with IgG subclass deficiencies involving subclass 2. Complement testing demonstrated normal values. Other possible causes of seizure onset were considered and assessed, including metabolic disturbances, medication-related provocation, and structural pathology. No alternative etiology more convincingly accounted for the overall presentation.

Given persistent seizures despite antiseizure therapy and concern for immune-mediated neurologic dysfunction, the patient was treated with intravenous methylprednisolone, followed by IVIG. Antimicrobial therapy was continued during IVIG treatment. Seizure frequency declined within one week of immunomodulatory treatment. By six weeks, she was seizure-free. At six-month follow-up, she remained seizure-free without antiseizure medication and continued on doxycycline, 100 mg twice daily.

## Discussion

This case series describes two previously healthy patients who developed new-onset or worsening seizures in temporal association with Lyme-associated illness and antimicrobial treatment, with subsequent clinical improvement following immunomodulatory therapy. The cases are notable for several shared features: evolving seizure disorders refractory to multiple antiseizure medications; serologic findings interpreted as supportive, but not definitive, for Lyme disease; immune laboratory abnormalities, including hypogammaglobulinemia and IgG subclass deficiency; and marked improvement after corticosteroids and IVIG. Together, these findings raise the possibility that infection-associated immune activation and neuroinflammation contributed to the neurologic presentations. However, they do not establish a direct causal relationship between *B. burgdorferi* infection and autoimmune epilepsy.

A key distinction in interpreting these cases is the difference between confirmed neuroborreliosis and the broader hypothesis of infection-triggered, immune-mediated seizures. We do not present these cases as evidence that direct infectious neuroborreliosis caused the observed seizures. Rather, we propose that Lyme-associated illness may have coincided with, or contributed to, an immune-mediated neurologic process. This distinction is especially important because evidence linking *B. burgdorferi *to autoimmune epilepsy remains limited, compared with more established paradigms, such as herpes simplex virus-associated autoimmune encephalitis [5].

The diagnostic interpretation of Lyme disease in these cases warrants caution. In both patients, immunoblot results demonstrated reactive bands, but did not meet CDC surveillance criteria for a positive IgG Western blot. This limitation is acknowledged explicitly. At the same time, CDC surveillance case definitions are intended for epidemiologic reporting and are not designed to determine whether an individual patient has Lyme disease, or to direct treatment decisions [6]. The serologic findings were therefore interpreted in conjunction with exposure history, symptom evolution, and the overall clinical presentation. Both patients also had histories suggestive of longstanding Lyme-associated illness, and interpretation of serologic testing in such settings can be challenging. Antibody responses may vary depending on the timing of infection, prior antimicrobial exposure, and individual host immune responses. These factors may complicate the interpretation of standard serologic testing and underscore the importance of clinical context.

The temporal worsening of seizures after initiation of antimicrobial therapy is also noteworthy. One possible explanation is an inflammatory response associated with microbial die-off, similar to a Jarisch-Herxheimer-type reaction. Such reactions are well described in spirochetal infections and are thought to involve transient inflammatory cytokine release following initiation of antimicrobial therapy [12]. Jarisch-Herxheimer reactions have been reported in Lyme disease, although they appear to be relatively uncommon, and seizures in Lyme neuroborreliosis are also uncommon, described primarily in isolated reports. Accordingly, this mechanism should be considered a plausible, but not definitive, explanation for the clinical deterioration observed here.

These cases also raise the broader question of how best to conceptualize the immune response. Immune activation does not necessarily equate with classical autoimmunity. The observed neurologic course may reflect infection-associated immune activation, neuroinflammation, or an immune-mediated seizure process that does not fit neatly into existing categories. For this reason, the terminology used in this report refers not only to autoimmune epilepsy, but also, more broadly, to infection-associated immune activation and neuroinflammation, reflecting the range of potential immune mechanisms considered in these cases. The relationship between infection, inflammation, and immune-mediated neurologic dysfunction in Lyme-associated illness is likely complex, rather than binary.

The neural autoantibody results should likewise be interpreted cautiously. In Case 1, a low-titer serum NMDAR antibody was detected on a cell-based assay, but confirmatory tissue-based immunofluorescence was negative. In Case 2, dopamine receptor D1 antibodies were elevated, with borderline anti-lysoganglioside GM1 and anti-tubulin antibodies. Some of these antibodies, particularly dopamine receptor antibodies, have been described in immune-mediated neurologic and neuropsychiatric syndromes, but their diagnostic specificity and validation in autoimmune epilepsy remain uncertain. Thus, the antibody findings are supportive of immune dysregulation, but are not independently diagnostic.

The immunologic abnormalities in both patients are also notable. Hypogammaglobulinemia, low IgA/IgG, IgG subclass deficiencies, and complement measurements were incorporated into the immune evaluation to better characterize host immune status. Although these abnormalities do not prove an autoimmune process, they may reflect altered immune regulation and may increase vulnerability to infection-associated immune dysfunction. IVIG was selected in part because it offered both immunomodulatory therapy and immunoglobulin replacement in the setting of documented immunoglobulin deficiency. More aggressive B-cell-depleting strategies were deferred, given concern for prolonged immune suppression in patients undergoing treatment for suspected infection-associated illness [15,16].

The clinical response to therapy is one of the most striking features of both cases. Seizures persisted despite multiple antiseizure medications and prolonged antimicrobial treatment, including intravenous therapy in Case 1. In both patients, antimicrobial therapy was continued during IVIG administration, yet substantial improvement followed immunomodulatory therapy. This pattern does not prove that infection was absent or irrelevant. Persistent infection cannot be definitively excluded, and corticosteroids can reduce inflammation, regardless of etiology. Nonetheless, the failure of standard antiseizure medications and the marked clinical improvement after corticosteroids and IVIG support consideration of an immune-mediated component to seizure pathogenesis.

Several limitations must be acknowledged. First, the sample size is extremely small, precluding broad conclusions. Second, Lyme disease testing did not meet CDC surveillance criteria for a positive IgG immunoblot, creating diagnostic uncertainty. Third, some of the neural autoantibody assays used have limited validation in autoimmune epilepsy. Fourth, the observed seizures could represent primary autoimmune epilepsy, or another inflammatory neurologic process temporally associated with antecedent infection, rather than a syndrome directly triggered by *B. burgdorferi*. Finally, although persistent infection could not be definitively excluded, the cases do not establish that continuing infection was the sole or primary driver of neurologic dysfunction.

Despite these limitations, the cases underscore an important clinical point: patients with treatment-refractory seizures may warrant evaluation beyond escalation of antiseizure medications alone. In selected patients - particularly those with inflammatory symptoms, relevant infectious exposures, or atypical disease progression - consideration of infectious and immune-mediated contributors may broaden the differential diagnosis, and influence management.

## Conclusions

These cases demonstrate a temporal association between Lyme-associated illness, infection-associated immune activation, antimicrobial therapy, and treatment-refractory seizures. The observed clinical pattern - limited response to multiple antiseizure medications, worsening after antimicrobial initiation, and subsequent improvement after corticosteroids and IVIG - raises the possibility that immune-mediated mechanisms contributed to the neurologic manifestations in these patients. However, the diagnostic evidence for Lyme disease was not definitive by CDC surveillance criteria; persistent infection cannot be fully excluded, and the small sample size precludes conclusions regarding causality. These observations should, therefore, be regarded as hypothesis-generating. They support maintaining a broad differential diagnosis and considering neuroimmune evaluation in selected patients with new-onset or refractory seizures temporally associated with infectious illness.
